# Characterization of extracellular spike waveforms recorded in wallaby primary visual cortex

**DOI:** 10.3389/fnins.2023.1244952

**Published:** 2023-09-08

**Authors:** Young Jun Jung, Shi H. Sun, Ali Almasi, Molis Yunzab, Hamish Meffin, Michael R. Ibbotson

**Affiliations:** ^1^Department of Biomedical Engineering, The University of Melbourne, Melbourne, VIC, Australia; ^2^National Vision Research Institute, Australian College of Optometry Carlton, Carlton, VIC, Australia; ^3^Department of Optometry and Vision Sciences, The University of Melbourne, Melbourne, VIC, Australia

**Keywords:** primary visual cortex, receptive fields, spike waveforms, marsupials, orientation selectivity

## Abstract

Extracellular recordings were made from 642 units in the primary visual cortex (V1) of a highly visual marsupial, the Tammar wallaby. The receptive field (RF) characteristics of the cells were objectively estimated using the non-linear input model (NIM), and these were correlated with spike shapes. We found that wallaby cortical units had 68% regular spiking (RS), 12% fast spiking (FS), 4% triphasic spiking (TS), 5% compound spiking (CS) and 11% positive spiking (PS). RS waveforms are most often associated with recordings from pyramidal or spiny stellate cell bodies, suggesting that recordings from these cell types dominate in the wallaby cortex. In wallaby, 70–80% of FS and RS cells had orientation selective RFs and had evenly distributed linear and nonlinear RFs. We found that 47% of wallaby PS units were non-orientation selective and they were dominated by linear RFs. Previous studies suggest that the PS units represent recordings from the axon terminals of non-orientation selective cells originating in the lateral geniculate nucleus (LGN). If this is also true in wallaby, as strongly suggested by their low response latencies and bursty spiking properties, the results suggest that significantly more neurons in wallaby LGN are already orientation selective. In wallaby, less than 10% of recorded spikes had triphasic (TS) or sluggish compound spiking (CS) waveforms. These units had a mixture of orientation selective and non-oriented properties, and their cellular origins remain difficult to classify.

## 1. Introduction

Neurons in the primary visual cortex (V1) fire action potentials (or spikes) when appropriate visual stimuli appear within their receptive fields (RFs). Analyzing spiking responses in relation to the visual stimulus has made it possible to determine the preferred visual features within the RFs of neurons in the pathway from retina to V1 in a range of species (Hubel and Wiesel, 1962, 1968; Rust et al., 2004; Huberman et al., 2006; Van den Bergh et al., 2010). In cat V1, most cells have elongated, orientation selective RFs, while the cells in the lateral geniculate nucleus (LGN), which provides V1’s main input, have non-orientation selective RFs to white noise (Smith et al., 1990; Gharat and Baker, 2017). Additionally, further information can be partially inferred from the waveform of a spike about the type of neuron being recorded, such as whether it is excitatory or inhibitory or whether it is from the soma of a local neuron or axon terminal of a neuron projecting from another area (Jia et al., 2019; Onorato et al., 2020). This makes it possible to correlate cell types with their RFs (Sun et al., 2021).

**Figure 1:**
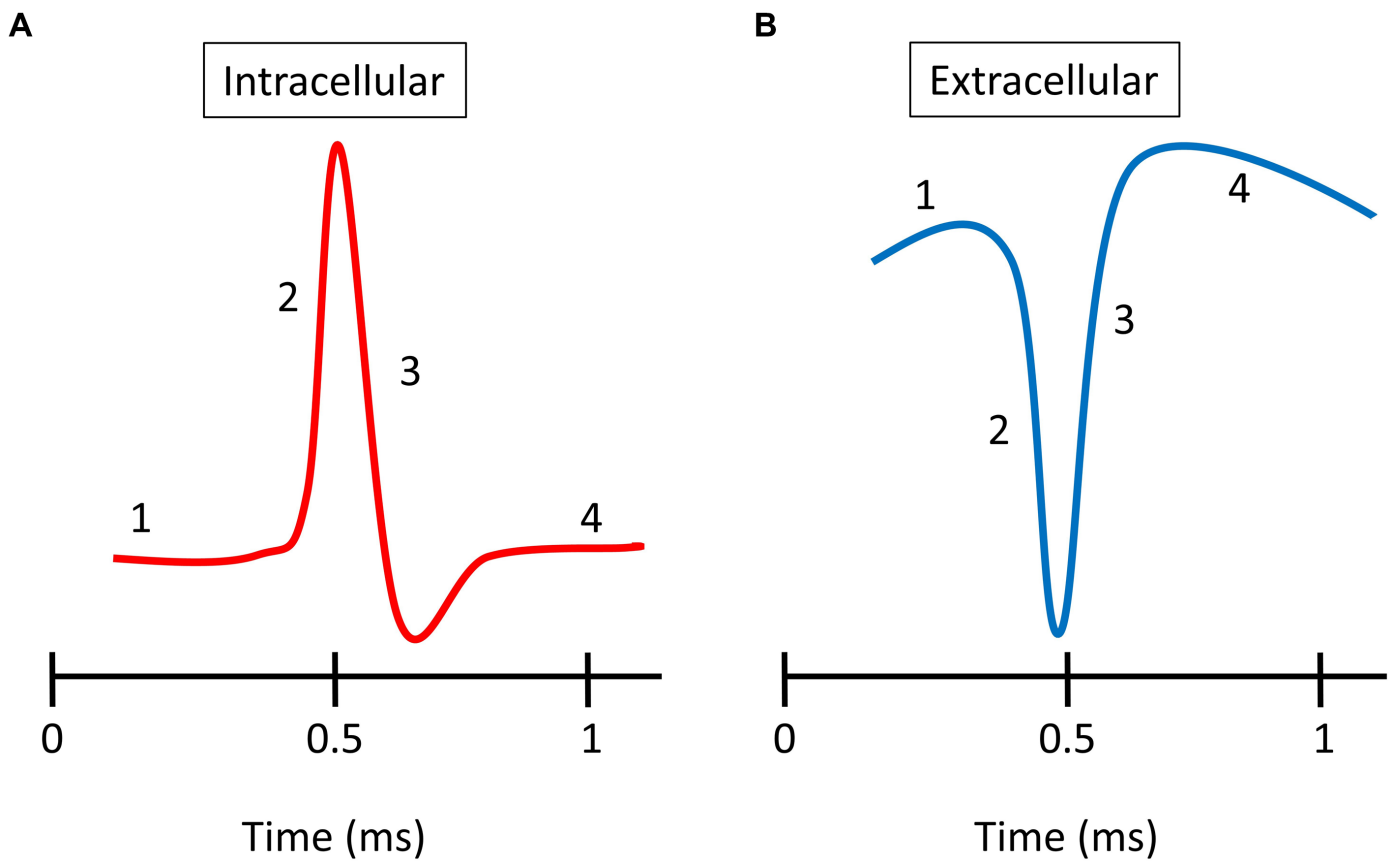
Spike waveforms. **(A)** Typical intracellular spike recorded from an axon. **(B)** Typical extracellular spike recorded in the somal region. There are four phases shown: (1) resting membrane potential; (2) depolarization; (3) repolarization; and (4) overshoot hyperpolarization.

Intracellularly, spike waveforms are classically described as having four different phases: (1) resting membrane potential; (2) depolarization when Na + channels open and Na + ions enter the cell; (3) repolarization when Na + channels close, K+ channels open, and K+ ions exit the cell; and (4) hyperpolarization when K+ channels close slowly (Figure 1). However, intracellular spike shapes can vary from this basic plan for a range of reasons, e.g., the type of neuron, the brain area, and the location of the electrode relative to the cell body (Dyball et al., 1991; Quirk and Wilson, 1999). Recordings from cortical cell bodies (somas) reveal a range of shapes that depend on the cell class and role (Mason and Larkman, 1990; Stuart et al., 1993). For example, broad spike-waveforms (regular spiking, RS) arise from excitatory neurons (often pyramidal cells), whereas narrow spike-waveforms (fast spiking, FS) originate from inhibitory neurons, e.g., basket and chandelier cells (Azouz et al., 1997; Ahmed et al., 1998; Henze et al., 2000).

Extracellular spike shapes are closely related to the changes in membrane potential observed during intracellular recording but with the polarity of the waveform reversed (Figure 1; Henze et al., 2000). As with intracellular waveforms, a number of extracellular spike shapes have been described. Sun et al. (2021) identified five distinct classes of extracellular spike waveforms in cat V1: regular spiking (RS), fast spiking (FS), triphasic spiking (TS), compound spiking (CS), and positive spiking (PS). The RS and FS cells have biphasic waveforms with dominant negative troughs that are followed by either a recovery to normal levels or a smaller positive peak. RS and FS waveforms have broad and narrow shapes, respectively. The RS and FS spikes have been described in all studied species and represent the most common types (Henze et al., 2000; Buzsaki and Draguhn, 2004; Gold et al., 2006, 2009; Gharat and Baker, 2017). TS waveforms have a pronounced first positive peak followed by a larger negative trough and then a second positive peak. CS waveforms are similar to TS waveforms, but they have the final positive peak >1 ms after the first peak. PS waveforms have a positive peak larger than their negative trough. The anatomical identity of the cells that generate TS and CS spikes remains unclear. However, Sun et al. (2021) argued that PS units in cat V1 are recordings from afferents from the lateral geniculate nucleus (LGN), as the single units with positive-dominant spikes had mostly non-oriented RFs and short latencies, as expected from LGN units. Several studies have previously reported PS waveforms (Gold et al., 2006, 2009; Robbins et al., 2013; Gharat and Baker, 2017). The significant positive peak is thought to occur when recording from distal regions of the axon (Gold et al., 2006). The different spatial locations relative to the cell body can reflect different extracellular spike waveforms. The amplitude of the negative trough, associated with depolarization (represented in amplitude-dependent color), decreases rapidly with distance from the cell body, and is associated with a relative increase of the 1st peak.

Here we report extracellular spike waveforms from single units recorded from V1 of an anaesthetized marsupial species, the Tammar wallaby, to compare with data from eutherian mammals (e.g., cats and monkeys). We then correlated the extracellular waveform classes with their associated spatial RFs, estimated using the nonlinear input model (NIM). Wallaby visual cortex is dominated by RS and FS units, which have mainly orientation-selective RFs. We also find that PS units in wallaby V1 have very short latencies and bursty behavior, supporting the notion that the recordings are from LGN axon afferents.

## 2. Materials and methods

### 2.1. Ethics approval and experimental procedures

Recordings were made from six male adult tamer wallabies (12–24 months), each weighing between 3.9–5.7 kg. The experiments were conducted using the facilities at the National Vision Research Institute (NVRI) in accordance with the National Health and Medical Research Council’s *Australian Code of Practice for the Care and Use of Animals for Scientific Purposes*. All experimental procedures were approved by the Animal Care Ethics Committee at the University of Melbourne (Approval ID: 1714178.1). For a more detailed description of the experimental procedures, find https://www.science.org/doi/10.1126/sciadv.abn0954. Note that the animals used in this project are the same animals as used in Jung et al. (2022).

### 2.2. Visual stimuli

Visual stimuli were generated with a ViSaGe visual stimulus generator (Cambridge Research Systems, Cambridge, UK) and displayed on a calibrated, gamma-corrected LCD monitor (ASUS VG248QE, 1,920 × 1,080 pixels, refresh rate 60 Hz, 1 ms response time) at a viewing distance of 57 cm. White Gaussian Noise (WGN) stimuli were used to estimate the spatial RFs of cortical cells. WGN images comprised of 90 × 90 pixels over 30° of the visual field, with its mean pixel value matched to the mean luminance of the display monitor. Each noise block comprised of 12,000 WGN images, which were each presented for 1/30 s. It was followed by a blank screen of the mean luminance, displayed for the same duration of 1/30 s. The blank screen was used to increase the overall response of the cell to the stimuli as it would minimize any temporal correlation in the responses.

### 2.3. Data recording

Extracellular recordings were conducted using NeuroNexus 32-channel multi-electrode arrays (MEAs). We used two MEAs; a single shank probe (6 mm total length; 1 × 32 sites spaced at 100 μm intervals for the lower 3.2 mm) or a four-shank probe (6 mm total length, 4 × 8 sites spaced at 100 μm intervals for the lower 0.8 mm). The arrays were vertically inserted into the cortex using a piezoelectric drive (Burleigh inchworm and 6,000 controller, Burleigh instruments, Rochester, NY). Extracellular signals from 32 channels were simultaneously acquired using a CerePlex acquisition system and Central software (Blackrock Microsystems, Salt Lake City, Utah) sampled at 30 kHz.

### 2.4. Spike waveform analysis

An automatic spike-sorting program called KiloSort (Pachitariu et al., 2016) was used to separate spikes from different neurons. The spike clusters were manually sorted for further verification using the graphical user interface phy (Rossant et al., 2016). See methods from Jung et al. (2022). We used each single unit’s mean extracellular spike waveform for all waveform analyses. To obtain the mean waveform, 10,000 random spike waveforms were collected from each SU from the raw data on the recording channel with the highest amplitude. These waveforms were extracted between 1 ms before and 2 ms after the spike time (in a window of −30 to 60 samples, where 0 is the minimum trough). We used the maximum number of spikes for SUs with less than 10,000 spikes (<10% of the total population). Collected spike waveforms were averaged, and the baseline was subtracted to make the new baseline equal to 0. The baseline was calculated as the mean of the first and last 10 samples of the extracted waveform. For each mean spike waveform, we measured five features: amplitude, peak-trough ratio, end-slope, duration, and 1^st^ peak-trough ratio.

#### 2.4.1. Amplitude

The amplitude was calculated as the difference between the maximum peak or the minimum trough and the baseline of the waveform. The maximum peak was used when the magnitude was larger than the minimum trough, and if the minimum trough was larger than the maximum peak, the minimum trough was used.

#### 2.4.2. Peak-trough ratio

The peak-trough ratio was calculated as the ratio between the unsigned amplitudes of the minimum trough and the following peak: i.e., the amplitude of the peak was divided by the amplitude of the minimum trough.

#### 2.4.3. End-slope

The end-slope was calculated as the gradient of the slope at 0.5 ms after the minimum trough.

#### 2.4.4. Duration

The duration was calculated as the time from the minimum trough and the following peak.

#### 2.4.5. 1^st^ peak-trough ratio

The 1^st^ peak-trough ratio was calculated as the ratio between the amplitude of the minimum trough and the previous peak: i.e., the amplitude of the 1^st^ peak was divided by the amplitude of the minimum trough.

### 2.5. Analysis of receptive fields

We used the nonlinear input model described by Almasi et al. (2020) to estimate the spatial RFs. This framework is an adaptation of the original model, introduced by McFarland et al. (2013), that estimates all model parameters simultaneously. We quantified the orientation selectivity of every filter with an OB index as follows:


$$OB=\frac{\left|\sum_{k}R_{k}\;\exp\;\left(i2\theta_{k}\right)\right|}{\sum_{k}R_{k}\;}$$

where $R_{k}$ represents the neuronal response at orientation $\theta_{k}$ , and $i=\sqrt{-1}$ . We adopted this measure for the amplitude spectrum of filters. $R_{k}$ represents the amplitude spectrum of the filter sampled at orientation $\theta_{k}.$ The number of spatial filters of each unit was systematically varied, and we evaluated the statistical significance of each filter using bootstrapping, which is a method commonly used in RF model evaluation (Rust et al., 2005; Touryan et al., 2005; Almasi et al., 2020). For more details see the methods section in Almasi et al. (2020).

### 2.6. Analysis of spiking properties

#### 2.6.1. Spike rate

The spike rate of a single unit was determined by counting the spikes across the entire duration of the recording and dividing it by the length of the recording.

#### 2.6.2. Response latency

The optimum response latency of a single unit was calculated from the post stimulus time histogram (PSTH). The PSTH was established from the stimulus onset (time = 0 ms) to the stimulus offset (time = 66.67 ms), and spikes were binned at 1/15 ms. The PSTH was smoothed by averaging over a sliding window of length 5 ms. Latency was determined as the point in time when the spike rate reached 15% above the minimum ongoing spike rate inside 0–66.67 ms.

#### 2.6.3. Burst index

The burst index was calculated as the ratio for all spikes (i.e., nBurst/nAll). Spikes were defined as bursty when the inter-spike interval before the first spike was more than 100 ms and when the subsequent spikes were within an inter-spike interval of less than 4 ms. This burst index calculation is adapted from Wang et al. (2006).

## 3. Results

### 3.1. Classification of extracellular spike waveform classes

**Figure 2:**
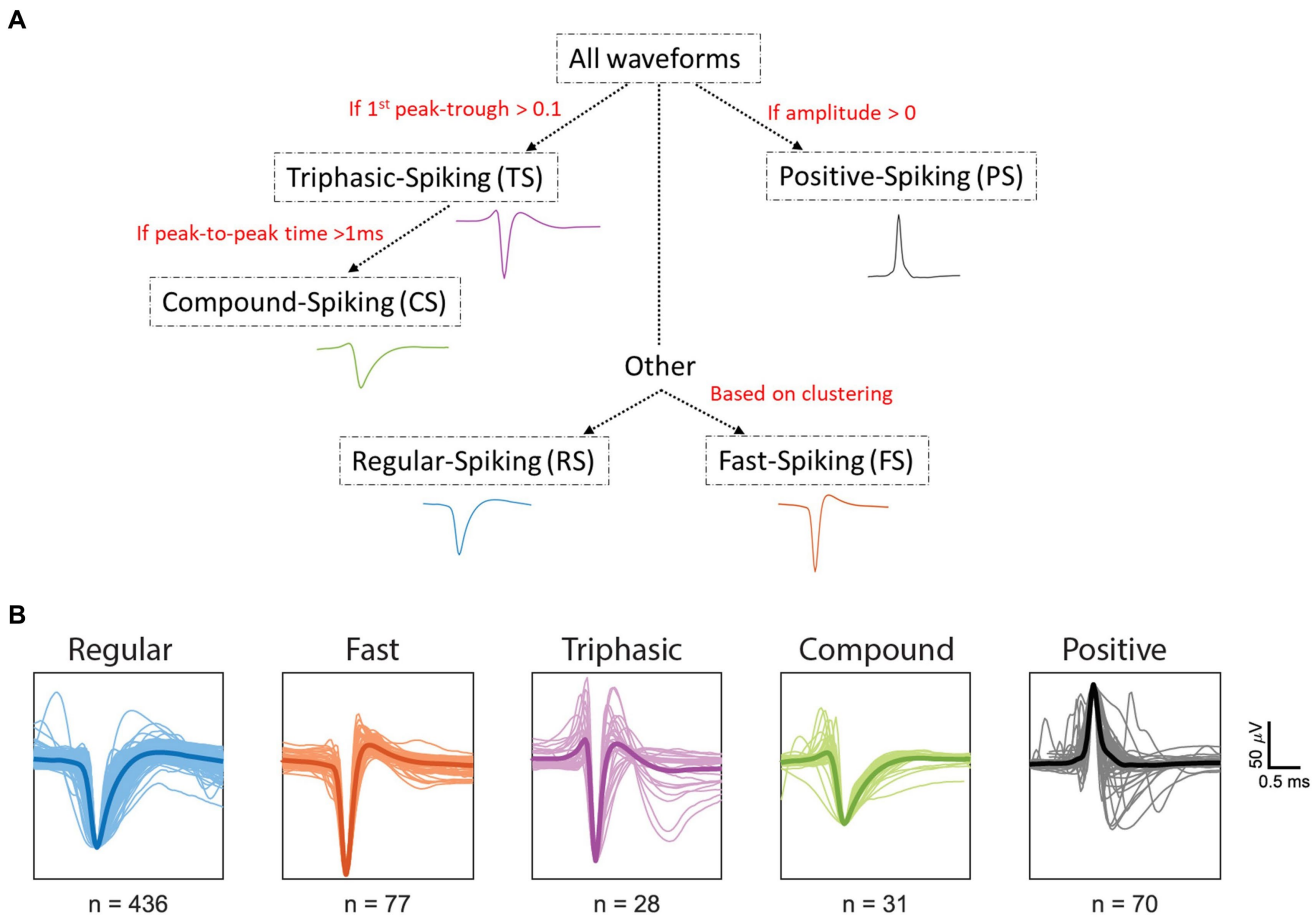
Classification of spike waveforms from all units. **(A)** Decision tree was used in classifying the spike waveforms. **(B)** Regular spiking (RS, blue), fast spiking (FS, orange), triphasic spiking (TS, purple), and compound spiking (CS, green) waveforms were normalized and aligned to the average minimum trough. Positive spiking (PS, black) waveforms were normalized and aligned to the average maximum peak. Dark lines within each waveform class represent the mean spike waveform. The X and Y axes for all units represent time (ms) and microvolts (μV), respectively. The spikes were collected between −1 ms to 1.5 ms from the minimum trough. Scale bars represent 0.5 ms (horizontal) and 50 μV (vertical).

We recorded from 642 single units (SUs) in the primary visual cortex of six anesthetized wallabies, of which 195 of the units were driven by white Gaussian noise (WGN) and had their spatial receptive fields (RFs) uncovered using the nonlinear neural input model (NIM). We found that wallaby RFs have up to 5 spatial filters: 56% with one significant filter, 20% two filters, 17% three filters, 4% four filters and 3% five filters. In wallaby, orientation selective units dominate (76%) and the percentages are similar regardless of the number of spatial filters. From the 642 units recorded, we identified five distinct waveform classes: regular spiking (RS), fast spiking (FS), triphasic spiking (TS), compound spiking (CS), and positive spiking (PS). The categorization was conducted in a serial manner using a decision tree (Figure 2A), in the same method previously used for cat spike waveforms (Sun et al., 2021). First, units were classified as PS if they had a positive peak greater than their negative trough, while the remaining units were classified into negative spiking (NS) waveform types. Second, units were classified as TS if the magnitude of the 1^st^ peak was equal to or greater than 10% of the following negative trough (but not larger than the trough). Third, TS units with particularly long waveforms (>1 ms from peak-to-peak) were classified as CS units. Fourth, the remaining NS units were classified as RS and FS using the end-slope. The details on the classification of waveforms will be further discussed below. Figure 2B presents spike waveforms for all units from the five distinct waveform classes. From left to right, we present RS (blue, 68%, *n* = 436), FS (orange, 12%, *n* = 77), TS (purple, 4%, *n* = 28), CS (green, 5%, *n* = 31) and PS (black, 11%, *n* = 70) units. The dark line shows the mean spike waveform for each type of spike. For each spike waveform, we measured the signal to noise ratio (SNR) by calculating the ratio of the peak-to-peak amplitude of the mean waveform to twice the standard deviation, as described previously by Kelly et al. (2007). The mean SNR over the SU population was 2.19 ± 1.10 (SD). The SNR for waveform classes are as follows: RS (mean ± SD =2.09 ± 1.09), FS (mean ± SD =2.69 ± 1.33), TS (mean ± SD =2.54 ± 0.98), CS (mean ± SD =2.26 ± 1.32), and PS (mean ± SD =2.08 ± 0.92).

### 3.2. Regular and fast spiking units

**Figure 3:**
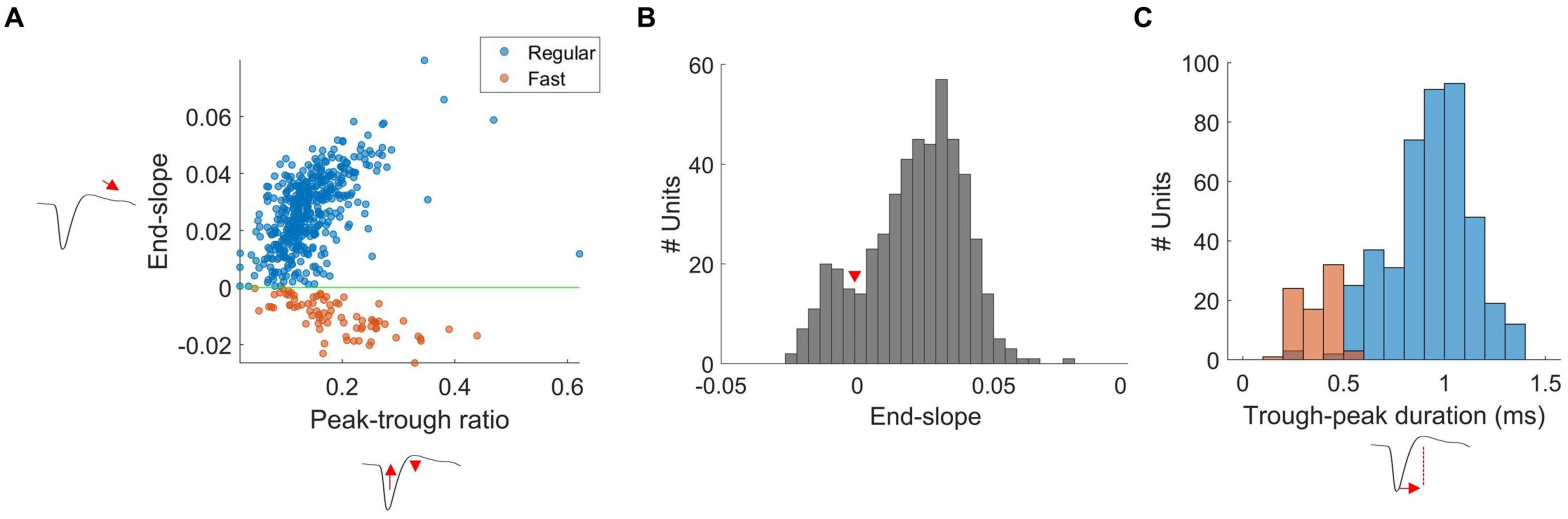
Separation of FS and RS units in wallaby V1. **(A)** Scatterplots of spike waveform shape parameters: end-slope and peak-trough ratio used to separate RS (blue) and FS (orange) units from all units were separated using an end-slope = 0 (green-line). The x and y axes denote peak-trough ratio and end-slope, respectively. **(B)** Histogram plot of the end-slope from all units. Bins are 0.005. The distribution of RS and FS units was significantly different from unimodality (Hartigan’s dip statistic: dip = 0.01, *p* < 0.05). **(C)** Histogram plots of the trough-peak duration (ms): RS (blue) and FS (orange) units from all units. Bins are in 0.1 ms.

RS and FS units had waveforms with a biphasic shape, with a strong negative trough followed by a smaller positive peak. RS and FS units were separated using end-slope and peak-trough ratio, as originally classified in the mouse visual cortex (Niell and Stryker, 2008). We measured the end-slope of the waveform at 0.33 ms after the trough and plotted against the peak-trough ratio (Figure 3A; i.e., the ratio between the normalized amplitude of the trough and the peak). The end-slope time of 0.33 ms after the trough provided one of the largest separations between clusters, as previously highlighted by Sun et al. (2021). RS and FS units were separated at an end-slope of zero. We classified spikes with an ascending slope (end-slope > 0) as RS, and spikes with a descending slope (end-slope < 0) as FS units, based on the Hartigan’s dip test (dip = 0.01, *p* < 0.05, Figure 3B). There were no units with an end-slope of zero.

**Figure 4:**
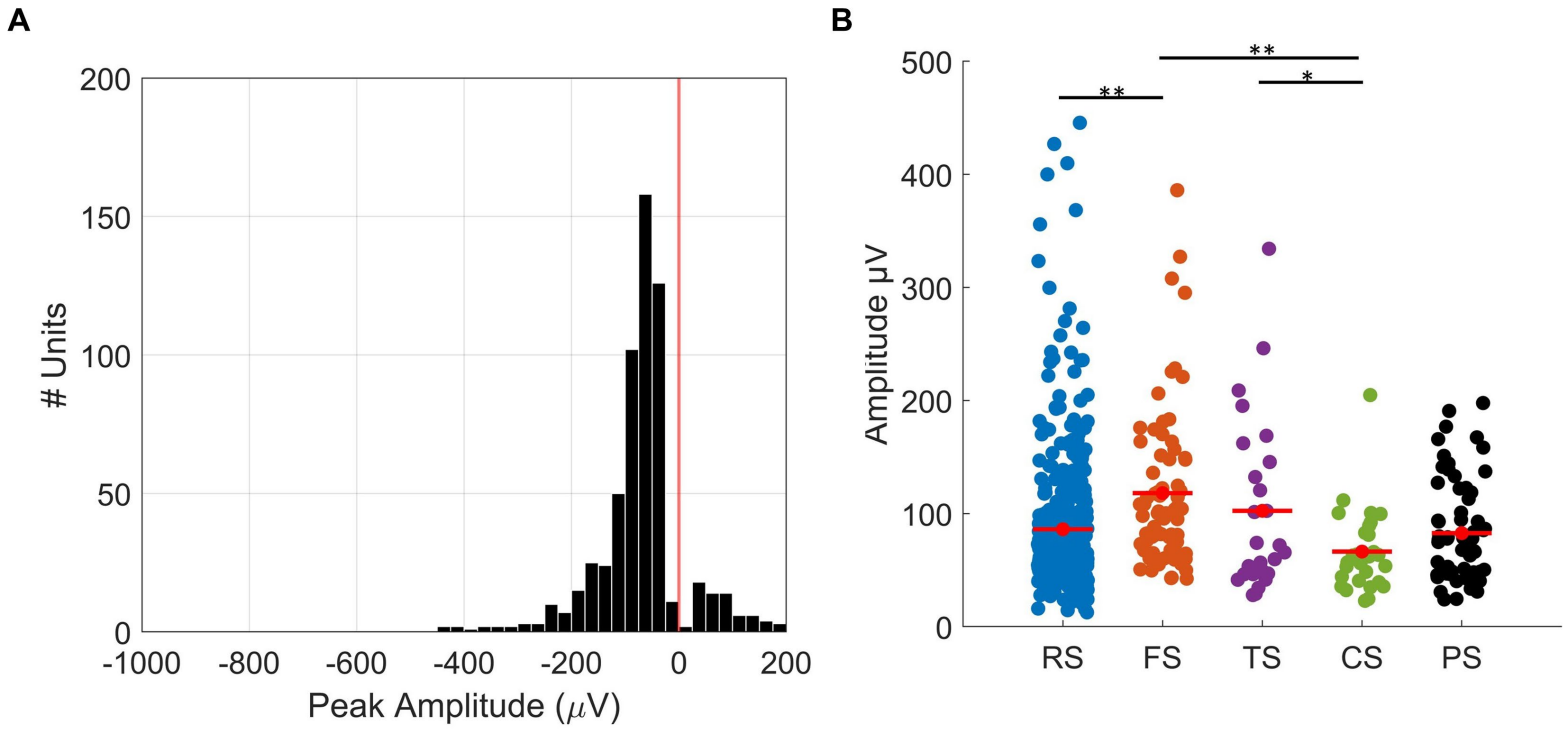
Peak spike amplitudes. **(A)** Histogram of the distribution of recorded maximum absolute amplitudes in 24 μV bins. The red line shows amplitude = 0, which is the threshold where the negative spikes (amplitude <0) were separated from positive spikes (amplitude >0). The mean amplitude from all negative spikes is −90.28 ± 64.68 μV (mean ± SD), and for positive spikes, the mean amplitude is 82.71 ± 44.19 μV (mean ± SD). **(B)** Plot showing the distribution of amplitudes of all units for each waveform class: RS (mean ± SD, blue, 86.12 ± 63.10 μV), FS (mean ± SD, orange, 118.08 ± 68.97 μV), TS (mean ± SD, purple, 102.43 ± 77.44 μV), CS (mean ± SD, green, 66.37 ± 36.95 μV), and PS (mean ± SD, black, 82.71 ± 44.19 μV) units. SEM is represented by red error bars. * and ** represent *p* < 0.05 and *p* < 0.01 (*t*-test), respectively.

RS units had a significantly smaller trough-to-peak ratio when compared to FS units (mean ± SD; RS: 0.14 ± 0.06, FS: 0.19 ± 0.08, *t*-test *p* < 0.01). Furthermore, we measured trough-peak duration (Figure 3C), as described by Thiele et al. (2016), to confirm the separation of RS and FS units. The histogram shows the distribution of trough-peak duration for RS (blue) and FS (orange) units. FS units had trough-peak durations predominately <0.5 ms (mean ± SD = 0.28 ± 0.21 ms), while in RS units, the ratio was >0.6 ms (mean ± SD = 0.78 ± 0.38 ms). We found that FS units had a significantly larger mean peak amplitude than that of the RS units (mean ± SD: FS =118.08 ± 68.97 μV, RS = 86.12 ± 63.10 μV, *t*-test, *p* < 0.01, Figure 4B).

#### 3.2.1. Positive spiking units

PS units had waveforms with a maximum peak larger than the minimum trough. Figure 4A presents a histogram of the distribution of peak amplitudes for all SUs. The red line (amplitude = 0) is the boundary that separates negative spikes (amplitude <0) from positive spikes (amplitude >0). The mean peak amplitude of PS units was 82.71 ± 44.19 μV (mean ± SD, Figure 4B), while the mean peak amplitude of all the negative spikes was 90.28 ± 64.68 μV (mean ± SD, Figure 4B).

#### 3.2.2. Triphasic spiking units

TS units had waveforms with initial peaks that had amplitudes smaller than the subsequent trough but with an initial peak amplitude greater than 10% of the minimum trough. The initial peak was followed by a large negative trough and usually a subsequent small positive peak. The mean initial positive peak from the TS units was 31 μV (30% of the minimum trough), which created a distinct triphasic shape different to those of the RS and FS units. The mean peak amplitude for TS units (102.43 ± 77.44 μV, Figure 4B) was similar to that of RS and FS units.

#### 3.2.3. Compound spiking units

CS units had waveforms with distinct triphasic shapes, with an initial positive peak, which was followed by a negative trough and a small positive peak. The CS units were separated from TS units by their significantly longer waveforms, which we quantified by measuring the peak-to-peak time (mean peak-to-peak time ± SD: CS = 1.4 ± 0.12 ms, TS = 0.45 ± 0.15 ms, *t*-test, *p* < 0.01). Of all the waveform classes, CS units had the smallest mean peak amplitude (66.37 ± 36.95 μV, Figure 4B).

### 3.3. Cell population

**Figure 5:**
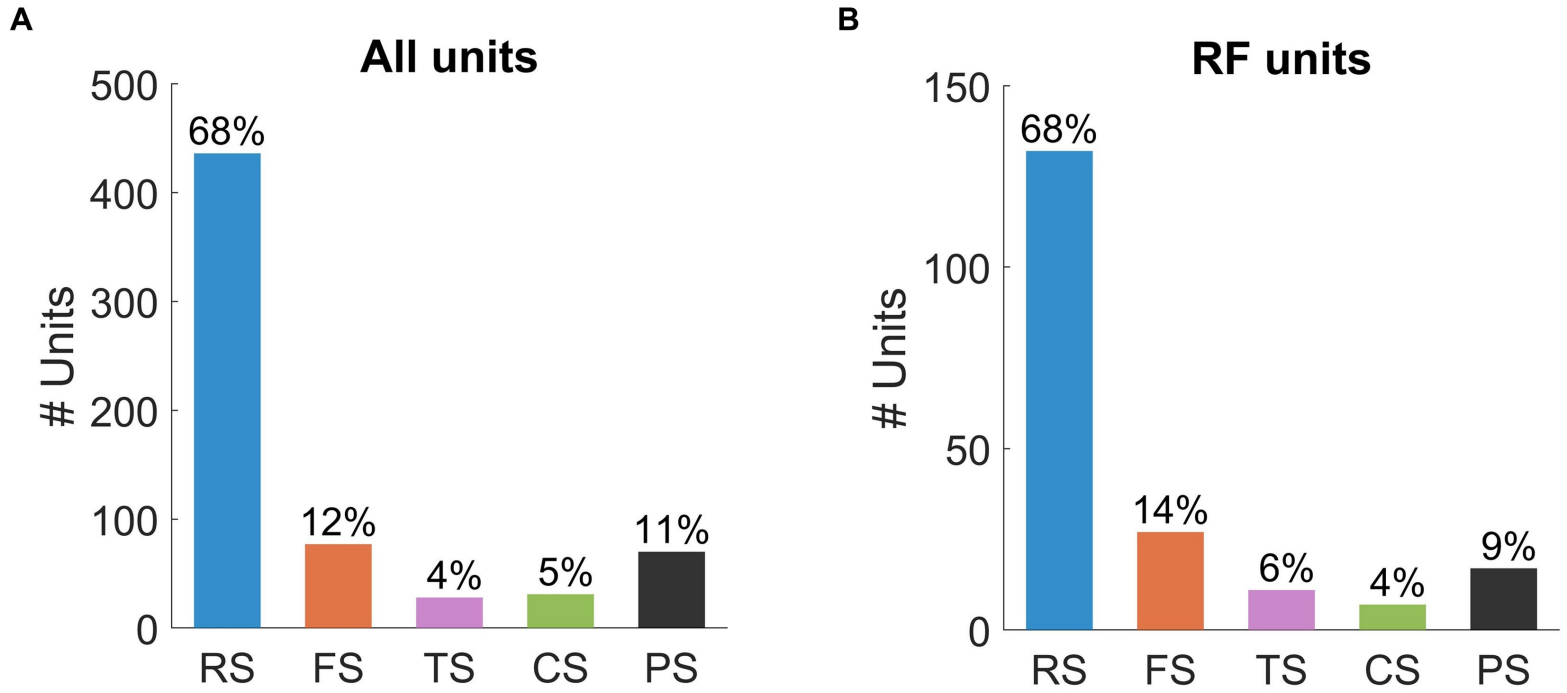
Population statistics for **(A)** all units and **(B)** units with RFs. Bars represent the number of units within each waveform class: RS (all units *n* = 436, RF units *n* = 132), FS (all units *n* = 77, RF units *n* = 27), TS (all units *n* = 28, RF units *n* = 11), CS (all units *n* = 31, RF units *n* = 7), PS (all units n = 70, RF units *n* = 17). The percentages of each subpopulation are presented above the bars.

Figure 5A summarizes the distribution of spike waveform types identified from all recorded units. The population was dominated by RS (*n* = 436, 68%) and FS units (*n* = 77, 12%). The ratio of RS units and FS units (68/12 = 5.7) identified in our population is higher than findings in previous extracellular studies in other species (2.2 for cat: Chen et al., 2015; 2.5 for macaque: Onorato et al., 2020; 4.1 for mouse: Niell and Stryker, 2008). The PS units (*n* = 70, 11%) were found to be the next largest group, though they formed a slightly lower proportion than those recorded in cat visual cortex (14%: Sun et al., 2021). Lastly, TS (*n* = 28, 4%) and CS units (*n* = 31, 5%) were found in the lowest proportions in the unit population.

We also classified spike waveforms from the population of units with RFs (*n* = 195, Figure 5B). The distribution of spike waveforms in this population was very similar to that in the population for all units. Of the 195 SUs, RS (*n* = 132, 68%) units were identified as the largest group, followed by FS units (*n* = 27, 14%). The ratio of RS units to FS units (132/27 = 4.9) was similar to that of the population for all units. The remaining waveform classes were all identified below 10% of the total population: TS units (*n* = 11, 6%), CS units (*n* = 7, 4%), and PS units (*n* = 17, 9%).

### 3.4. Correlating spike waveforms with receptive field structure

**Figure 6:**
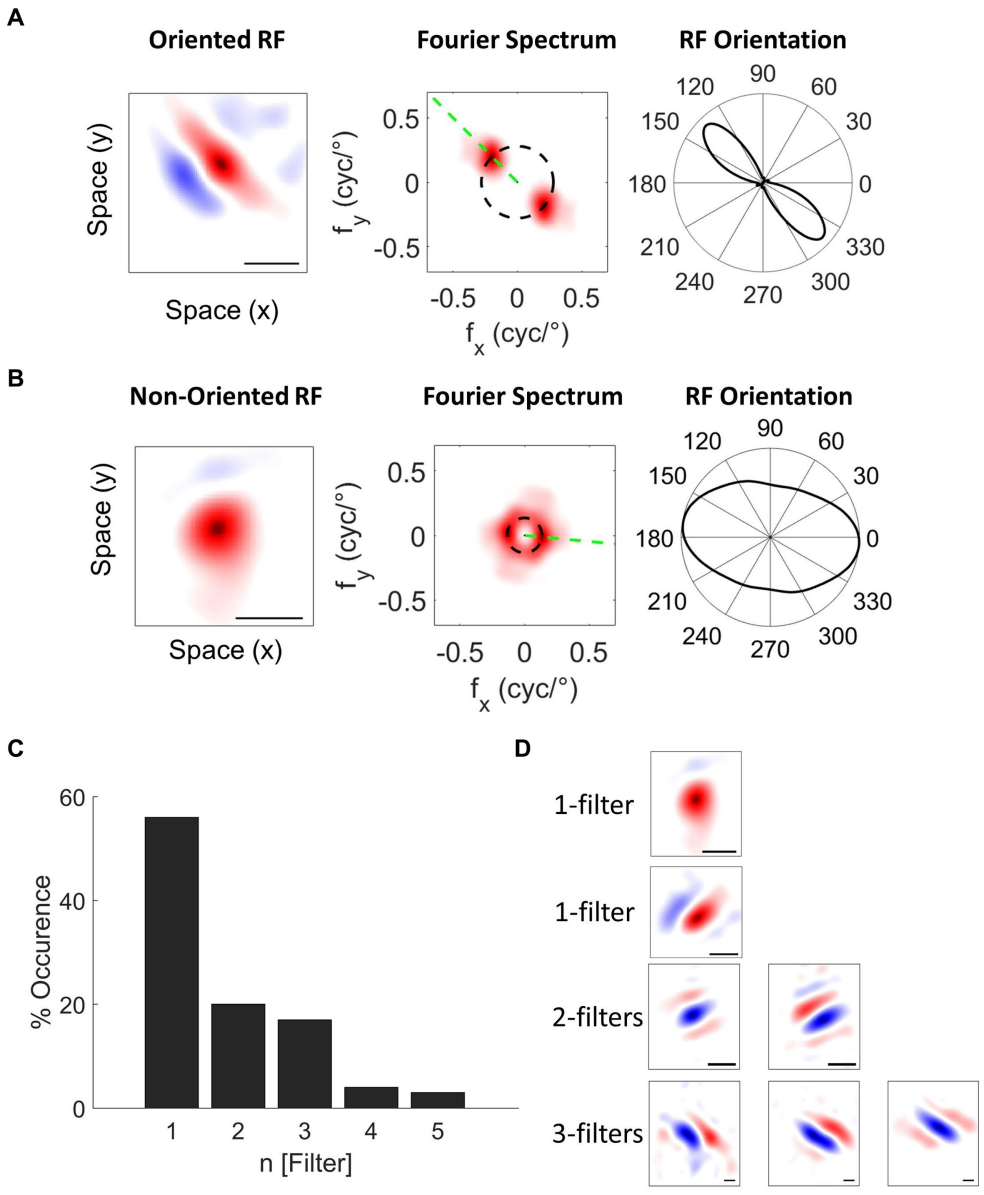
Classification of Spatial RFs. Example single units with **(A)** oriented and **(B)** non-oriented RF filters. The black scale bar indicates 1° of visual field. 2D Fourier amplitude spectrum of the RF filters. The red color intensity indicates the amplitude of the Fourier spectrum. The black dashed circle indicates the preferred spatial frequency of the filter. The green dashed line indicates the preferred orientation of the filter. Right panels: orientation tuning polar plots obtained by sampling the amplitude spectrum at the preferred spatial frequency OS of cells is quantified with an orientation bias (OB) index, which ranges from 0 (no selectivity) to 1 (narrow selectivity). **(C)** Distribution of the number of filters for single units recorded in wallaby V1 using the NIM. **(D)** Example single units with different RF filters.

From 195 well-isolated single units, we classified the units into oriented (76%, Figure 6A) and non-oriented (24%, Figure 6B) RF types using the orientation bias (OB) index calculated from neuronal responses to WGN (Leventhal et al., 2003; Talebi and Baker, 2016; Jung et al., 2022). We classified single units as non-orientation selective when the OB index ≤0.2. For single units with more than one filter, the amplitude of the 2D Fourier spectrum for each filter was normalized and then averaged to obtain the OB. Figure presents the proportion of oriented (blue) and non-oriented (yellow) RF units for each spike waveform type. In this plot, the cells are divided into oriented and non-oriented regardless of the number of filters. RS and FS units have mainly orientation-selective filters (RS: 80% oriented and 20% non-oriented; FS: 71% oriented and 29% non-oriented). TS and CS units have more oriented RFs than non-oriented RFs (TS: 73% oriented and 27% non-oriented; CS: 86% oriented and 14% non-oriented). Conversely, PS units have contain well-balanced portions of oriented and non-oriented units (PS: 53% oriented RFs and 47% non-oriented RFs). See Supplementary Figure 1 for the distribution of OB values for all the waveform types.

**Figure 7:**
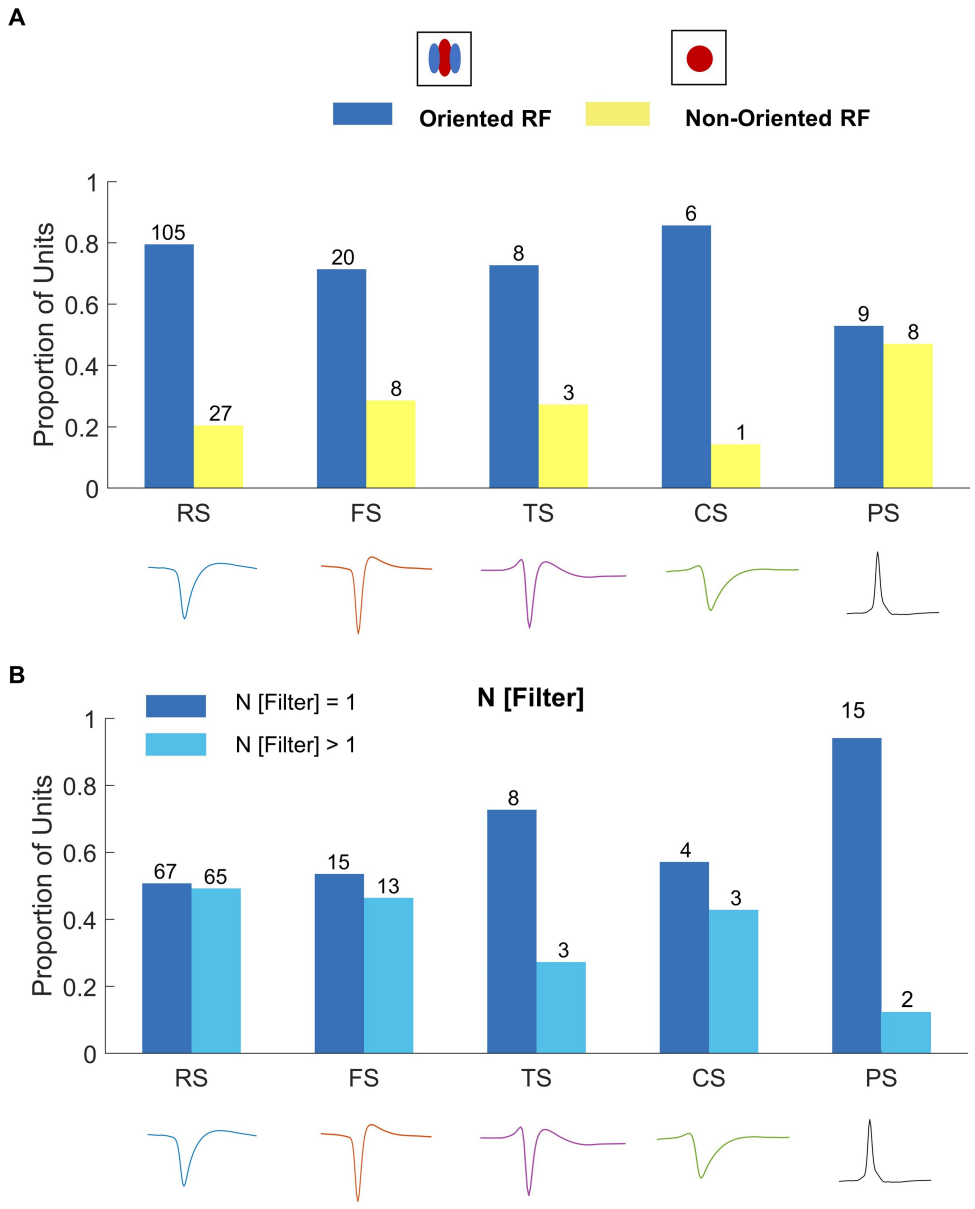
**(A)** Correlating spike waveform classes to oriented and non-oriented RFs. The legend above shows the RF types: oriented RF (blue) and non-oriented RF (yellow). RS and FS units are predominately oriented (RS: 67% oriented RF, *n* = 88; 33% non-oriented RF, *n* = 44; FS: 71% oriented RF, *n* = 20; 29% non-oriented RF, *n* = 8). TS, CS, and PS units have similar proportions of oriented RFs and non-oriented RFs (TS: 55% oriented RF, *n* = 6; 45% non-oriented RF, *n* = 5; CS: 57% oriented RF, *n* = 4; 42% non-oriented RF, *n* = 3; PS: 41% oriented RF, *n* = 7; 59% non-oriented RF, *n* = 10). **(B)** Correlating spike waveform classes to the proportion of single filter (blue; N [Filter] = 1) and multiple filter (light blue; N [Filter] > 1) units for each waveform type. RS, FS, and CS units have similar proportions of single and multiple filter units (RS: 51% single filter, *n* = 67, 49% multiple filters, *n* = 65; FS: 54% single filter, *n* = 15; 46% multiple filters, *n* = 13; CS: 57% single filter, n = 4, 43% multiple filters, *n* = 3). TS and PS units are dominated by single filter (TS: 73% single filter, *n* = 8, 27% multiple filters, *n* = 3; PS: 88% single filter, *n* = 15, 6% multiple filters, *n* = 2).

We also characterized nonlinearities in RF units based on the number of spatial filters (N Filter). Using a nonlinear input model of RFs, it is possible to extract the structure of the spatial filters that created each unit’s feature selectivity. A spatial filter in this context describes the spatial features in the image that generate responses from a given cell. Where cells have more than one filter, their nonlinear feature selectivity and invariance grows exponentially with the number of filters. Wallaby RFs have up to 5 spatial filters: 56% with one significant filter, 20% two filters, 17% three filters, 4% four filters and 3% five filters (Jung et al., 2022; Figures 6C,D). Figure 7B indicates that the distribution of RF units with single and multiple filters occurs in similar proportions for both RS and FS waveform classes (RS: 51% single filter and 49% multiple filters; FS: 54% single filter and 46% multiple filters).TS and CS units have greater proportions of RF units with single filters (TS: 73% single filter and 27% multiple filters; CS: 57% single filter and 43% multiple filters, Figure 7B). Likewise, 88% of PS units have a single spatial filter (Figure 7B).

### 3.5. Spiking characteristics

**Figure 8:**
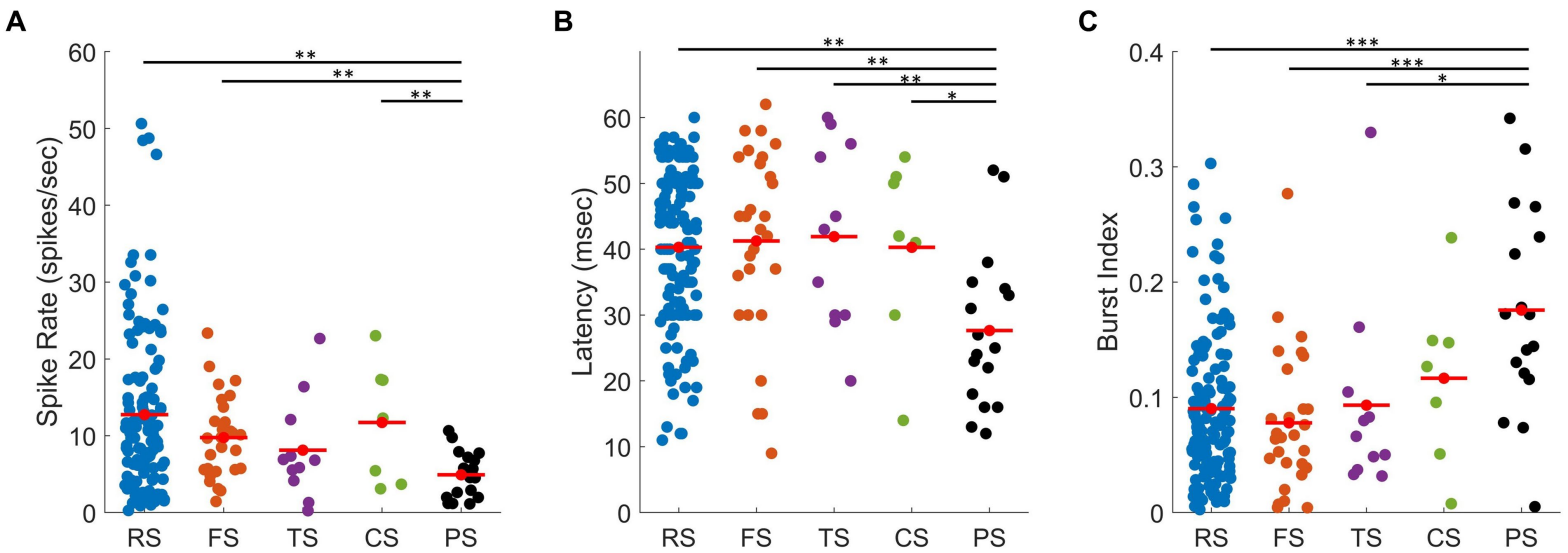
Scatter plots showing spiking characteristics of all units for each waveform class. **(A)** Spike rate of units with measured RFs: RS (blue, mean ± SD: 12.75 ± 10.43 spks/s), FS (orange, mean ± SD: 9.77 ± 5.34 spks/s), TS (purple, mean ± SD: 8.12 ± 6.60 spks/s), CS (green, mean ± SD: 11.73 ± 7.83 spks/s), and PS (black, mean ± SD: 4.92 ± 3.08 spks/s) units. **(B)** Latency, calculated by setting a 15% threshold from baseline to peak of the unit’s corresponding PSTH: RS (blue, mean ± SD: 40.29 ± 12.02 ms), FS (orange, mean ± SD: 41.25 ± 14.17 ms), TS (purple, mean ± SD: 41.91 ± 13.97 ms), CS (green, mean ± SD: 40.30 ± 14.13 ms), and PS (black, mean ± SD:27.65 ± 11.93 ms) units. **(C)** Mean burst index, i.e., ratio between burst spikes and all spikes: RS (blue, mean ± SD: 0.09 ± 0.06), FS (orange, mean ± SD: 0.08 ± 0.06), TS (purple, mean ± SD: 0.09 ± 0.09), CS (green, mean ± SD: 0.12 ± 0.07), and PS (black, mean ± SD: 0.18 ± 0.09) units. *, **, and *** represent *p* < 0.05, *p* < 0.01, *p* < 0.001 (*t*-test), respectively.

For each waveform class, several spiking characteristics were examined, i.e., spike rate, burst index, and response latency (Figures 8A–C). These spiking characteristics were chosen as their mean values differ between cortical and thalamic neurons. In this section, we only analyse the population of units with characterized RFs (*n* = 195). Figure 8A presents the average spike rate from each waveform class. PS units have the lowest average spike rate of all waveform classes (mean ± SD: 4.92 ± 3.08 spks/s). Their spike rates were significantly lower than RS (12.75 ± 10.43 spks/s, *t*-test: *p* < 0.01), FS units (mean ± SD: 9.77 ± 5.34 spks/s, *t*-test: *p* < 0.01), and CS units (mean ± SD: 11.73 ± 7.83 spks/s, *t*-test: *p* < 0.01), but not significantly lower than TS (mean ± SD: 8.12 ± 6.60 spks/s, *t*-test: *p* = 0.09).

Figure 8B presents the latency (i.e., the average time required to respond to stimulus onset) for each waveform class. The latency for each unit was calculated from the PSTH (as described in the Methods). PS units had the shortest latency (mean ± SD:27.65 ± 11.93 ms), which was significantly shorter than RS (mean ± SD: 40.29 ± 12.02 ms, *t*-test: *p* < 0.01), FS units mean ± SD: 41.25 ± 14.17 ms, *t*-test: *p* < 0.01), TS units (mean ± SD: 41.91 ± 13.97 ms, *t*-test: *p* < 0.01), and CS units (mean ± SD: 40.30 ± 14.13 ms, *t*-test *p* < 0.05). Short latencies are usually associated with the thalamic input in cat cortex, as they respond to visual input earlier than the cortical cells (Clay Reid and Alonso, 1995; Usrey et al., 2000; Alonso et al., 2001).

The burst index is indicative of how short the time interval is between the spikes. Thalamic cells are known to spike in bursts more often as compared to cortical cells (Guido and Weyand, 1995; Ramcharan et al., 2000). The burst index ranges from 0 to 1, representing low to high levels of bursting, respectively. Figure 8C presents the average burst index (i.e., the ratio of burst spikes over all spikes) for each waveform class. PS units had the highest proportion of bursting spikes mean ± SD: 0.18 ± 0.09), which were significantly higher than RS (mean ± SD: 0.09 ± 0.06, *t-*test: *p* < 0.001), FS units (mean ± SD: 0.08 ± 0.06, t-test: p < 0.001), TS units (mean ± SD: 0.09 ± 0.09, *t*-test: *p* < 0.05 and not significantly higher than CS units (mean ± SD: 0.12 ± 0.07, *t*-test: *p* = 0.1).

## 4. Discussion

### 4.1. Classification of extracellular spike waveform classes

We categorized spikes into five extracellular waveform classes: regular spiking (RS), fast spiking (FS), triphasic spiking (TS), compound spiking (CS), and positive spiking (PS). In RS and FS waveforms, the negative trough followed by a positive peak is established by the opening and closing of Na + and K+ channels, respectively (Henze et al., 2000). The dominant negative troughs observed in wallaby RS and FS waveforms provide compelling evidence that the recordings were made close to somas (Gold et al., 2006). In our study, we separated RS and FS units based on the difference in spike durations. In rodents, this difference has been understood to be the result of the difference in the level of expression of Na+ and K+ channels (Martina and Jonas, 1997; Martina et al., 1998); the spike duration in FS waveforms is shorter due to the faster repolarizations of Kv1 and Kv3 channels. Additionally, broad spike waveforms have been observed in pyramidal and spiny stellate neurons (i.e., excitatory neurons), whereas narrow spike waveforms have been observed in basket and chandelier interneurons (i.e., GABAergic inhibitory neurons) in rodent and cat V1 (Barthó et al., 2004; Trainito et al., 2019). However, the classification of RS and FS spike waveforms based on spike duration is not an absolute measure: recent studies in monkey cortex observed some pyramidal cells with narrow-waveform spikes (Ichinohe et al., 2004; Constantinople et al., 2009; Vigneswaran et al., 2011; Onorato et al., 2020). Extracellular spike duration also depends on the distance from soma to recording site, which will also affect RS and FS classification (Gold et al., 2006). However, this is the only such report, so the generality of this finding is hard to judge.

In contrast to RS and FS waveforms, TS, CS, and PS waveforms were found to have robust positive initial peaks (Figure 2), which are most likely due to a mixed-ion capacitive current that increases in amplitude as the recording site increases in distance from the soma (Figure 2; Gold et al., 2006). This is the case for dendritic spikes (Henze et al., 2000; Gold et al., 2009; Jia et al., 2019) and axonal spikes (Raastad and Shepherd, 2003; Blanche et al., 2005; Lewandowska et al., 2015). This phenomenon is consistent with models of current sources and sinks from extracellular spikes that propagate through an isolated axon, most likely due to differences in morphology and ion channels (Barry, 2015). The slow and small spikes associated with CS waveforms are most likely due to the large distance between the recording site and the soma and the consequent contribution of distal sources to the generation of the action potentials (Gold et al., 2006). In our cell population, we did not find any PS waveforms that exceeded 100 μV, which is consistent with Sun et al.’s (2021) findings in cat V1. However, this is in contrast to Gold et al.’s (2009) study in cat cortex that recorded larger extracellular positive spikes (>500 μV) with orientation selective RFs, which they named high-amplitude positive spikes (HAPS). Gold et al. (2009) believe that the HAPS may be recordings from the somas of large L5 pyramidal neurons undergoing distal dendritic initiation. The amplitude of any spike increases with the size of the soma and apical trunk, which might explain the large positive spikes of the HAPS (Gold et al., 2009). A possible explanation for the absence of HAPS in our data is that our recording sites did not typically reach L5 pyramidal neurons, as most recordings were made in the supragranular or granular layers (i.e., layers 1–4).

### 4.2. Origins and relative proportions of RS and FS spikes

The great majority of units recorded in this project (80%) were RS and FS units, with a heavy bias towards RS units (68% of all units were RS). The value of 68% RS units is far higher than the overall percentage of 53% found using identical recording apparatus in cat cortex (Sun et al., 2021), suggesting a real difference between species. The great majority of RS and FS units are orientation-selective, suggesting that several processing steps have occurred before them in the visual processing hierarchy. Extensive prior work by others has shown that it is very likely that RS and FS units, respectively, correspond to excitatory (i.e., pyramidal and spiny stellate) and inhibitory cortical cells (i.e., basket and chandelier; Barthó et al., 2004; Mitchell et al., 2007; Niell and Stryker, 2008; Mruczek and Sheinberg, 2012). In cat cortex, 72% of recorded units were either RS or FS, which matches quite well with the combined 80% in wallaby (Sun et al., 2021). However, it is interesting that the ratio of RS to FS waveforms in wallabies (RS/FS = 5.7) is much higher than ratios reported in other mammalian species (cat: RS/FS = 2.2; Chen et al., 2015; monkey: RS/FS (non-bursty) = 2.5; Onorato et al., 2020; mouse: RS/FS =4.1 Niell and Stryker, 2008). Given that we recorded from quite a large cell population (642 single units), the percentages for the more common units are likely quite reliable, suggesting that wallabies have a lot more cells producing RS spikes. This implies that there might be a higher proportion of excitatory cells in wallaby compared to commonly used eutherian mammals. It is hoped that recordings can be made from several marsupial cortices in the future to quantitatively assess whether cells with RS properties dominate in all marsupial cortices. If so, this would mark a distinct difference compared to Eutherian cortex. The fact that the cat RS/FS ratio was obtained using the same equipment in the same laboratory argues against a simple method-related difference.

The large over-representation of RS units in our wallaby recordings raises a more significant limitation associated with using extracellular spike waveforms to separate inhibitory and excitatory cells. It has been previously reported that some inhibitory cells display regular spiking waveforms that are indistinguishable from excitatory cells; namely basket cells that express cholecystokinin and vasoactive intestinal peptide (Wang et al., 2002; Zaitsev et al., 2009). Moreover, some studies have reported that some PV-expressing neurons can show regular spiking patterns, although the majority are FS in most species (50–74%: rat: Kawaguchi and Kubota, 1997; cat: Schwark and Li, 2000; mouse: Blatow et al., 2003; monkey: Krimer et al., 2005; human: Markram et al., 2004). It is possible that the outlier groups of RS inhibitory cells are larger in wallabies, and some RS units identified in this study might be inhibitory in nature.

### 4.3. Origins of other spike types

Positive spikes (PS) have been associated with recordings from the distal regions of axons (Bakkum et al., 2013; Pan et al., 2013; Lewandowska et al., 2015; Sun et al., 2021; Sibille et al., 2022). In cat primary visual cortex, it was found that 80% of PS units had non-orientation selective, blob-like RFs, low latencies, and bursty spiking properties (Sun et al., 2021). Based on these observations, it was proposed that the PS unit recordings in cat cortex might have arisen from LGN afferents. Is this also the case in wallaby cortex?

If the PS units in wallaby are LGN axons or axon afferents, why is it that only 49% have non-oriented RFs, while in cat, the great majority (78%) have non-oriented RFs (Sun et al., 2021)? One explanation might be that orientation selectivity is more developed in wallaby LGN than in cat LGN. Previous studies in cats defined non-oriented LGN cells as those that have an OB index <0.2 (Rosenberg et al., 2010; Gharat and Baker, 2017). We find that 70% of PS units have an OB index <0.3, which shows that most of the PS units are non-oriented or have only mild orientation biases. Consistent with our findings, in macaque V1 Gur et al. (1999) recorded small-amplitude positive spikes that were slightly less tuned to orientation than the negative spikes, but more selective to orientation than LGN cells. In their analysis of monkey cortex, they hypothesized that the small-amplitude positive spikes represented an intermediate processing stage in the evolution of orientation selectivity within the visual hierarchy. In cat, orientation selectivity largely emerges at the level of the synapses from LGN afferents (Scholl et al., 2013; Vidyasagar et al., 2015). Alternative to this classic convergence model of Hubel and Wiesel (1962), several studies have proposed that orientation selectivity in V1 emerges from the sharpening of orientation-biased LGN inputs (Vidyasagar and Sigüenza, 1985; Xu et al., 2002; Kuhlmann and Vidyasagar, 2011). Kuhlmann and Vidyasagar (2011) proposed that simple cells might receive excitatory input from orientation-biased LGN cells and inhibitory input from non-oriented LGN cells. They suggest that orientation selectivity may be driven by the interaction between excitatory and inhibitory inputs from the LGN cells, combined with recurrent cortical excitation and inhibition. Such a scheme is a possibility in the wallaby, but an extensive, quantitative investigation of wallaby LGN is required before conclusions can be drawn.

While the existence of orientation selectivity in many wallaby PS units could be used to argue against them being LGN axons, two other factors point strongly towards the PS recordings arising from LGN axons. First, the PS units had significantly shorter response latencies (28 ms) than other cortical recordings (RS and FS units: respectively, 40 and 41 ms), which is expected for cells earlier in the visual pathway (Jin et al., 2011; Takemura et al., 2020). Second, the PS units had a higher proportion of bursty responses than other cell types. In many species, LGN units have been found to have far greater burstiness than V1 units (Weyand et al., 2001; Bezdudnaya et al., 2006; Alitto et al., 2011). This is consistent with what has been reported in cats, where the PS units had the highest burst responses of all spike types recorded in V1 (Sun et al., 2021). However, an unusual feature of the PS units in wallabies was their low spike rates. In cats, the PS units were reported to have the highest spike rates of all unit types in V1 (Sun et al., 2021). In other species, LGN units also tend to have high spikes rates (macaque: Martinez-Conde et al., 2002; marmosets: Pietersen et al., 2017; cat: Yeh et al., 2003; mouse: Durand et al., 2016). While speculative at present, our cortical recordings suggest that marsupial LGN has relatively low spike rates compared to other mammals. Only a quantitative study of LGN in a range of marsupial species will be able to answer this question definitively.

In further support of the notion that PS units might be LGN afferents, there are two properties of PS cells that set them apart from the other cell types. First, when using the NIM filter analysis, a high proportion of PS units with recovered RFs were characterized as having linear summation properties (PS = 88%, single filter), as expected from the early stages of the visual processing pathway (Alonso et al., 2001; Ghodrati et al., 2017). X-cells in cat retina and LGN have linear summation properties (Enroth-Cugell and Robson, 1966; Shapley and Victor, 1978). PS units in cat V1 (area 17) were also found to have high proportions of linear summation characteristics, as expected if they were the afferents of LGN X-cells (Sun et al., 2021). Interestingly, while the NIM analyses support the notion of linear X-like receptive fields in many of PS units in wallaby V1 (PS = 88%, single filter), 12% of PS units had nonlinear receptive fields. In addition to the X-cells in cat retina and LGN, there are Y-cells (Enroth-Cugell and Robson, 1966; Shapley and Victor, 1978), which have very nonlinear summation properties (Rosenberg and Issa, 2011). It is likely that some of the recordings from wallaby PS units are from Y-cell LGN afferents. In cat, the input to Area 17 is primarily from X-cells, while that to Area 18 is primarily from Y-cells (Stone and Dreher, 1973; Friedlander et al., 1979). It is not known definitively whether there is both X- and Y-cell input to wallaby V1, as in primates (Shapley and Victor, 1981; Kaplan and Shapley, 1982; Solomon et al., 2002; Xu et al., 2002), or whether some segregation of the pathways occurs, as in cats. Our data implies that there is likely a mixed input from X and Y cells to wallaby V1.

Due to the low numbers of single units that had TS and CS waveforms (6 and 4%), it is difficult to draw strong conclusions on where their somas originate, but it is likely that TS and CS units are recordings from a mixture of cells with local cortical- or thalamic origin. This is because they have relatively even proportions of oriented and non-oriented RFs, which is consistent with what has been reported in cat cortex (Sun et al., 2021). They also have a higher proportion of bursty responses than the RS and FS waveforms, which is indicative of LGN cell responses. While the TS and CS waveforms have similar proportions of RF types, we have left them as separate classes due to the unusual shape, low amplitude, and slow waveforms of CS units. This CS waveform has only recently been described in cat cortex (Sun et al., 2021). As mentioned above, the modern sampling methods, i.e., MEAs and spike-sorting algorithms, may reveal these low amplitude spikes more readily than previous methods.

## Data availability statement

The raw data supporting the conclusions of this article will be made available by the authors, without undue reservation.

## Ethics statement

The animal study was approved by Animal Care Ethics Committee the University of Melbourne. The study was conducted in accordance with the local legislation and institutional requirements.

## Author contributions

MI and YJ: conceptualization and writing (original draft). YJ, SS, AA, HM, and MI: methodology. YJ, AA, SS, MY, HM, and MI: investigation. YJ and SS: visualization. MI: funding acquisition. MI and HM: supervision. MI, YJ, AA, SS, MY, and HM: writing (review and editing). All authors contributed to the article and approved the submitted version.

## Funding

This work was supported by the Australian Research Council Centre of Excellence for Integrative Brain Function (Grant CE140100007).

## Conflict of interest

The authors declare that the research was conducted in the absence of any commercial or financial relationships that could be construed as a potential conflict of interest.

## Publisher’s note

All claims expressed in this article are solely those of the authors and do not necessarily represent those of their affiliated organizations, or those of the publisher, the editors and the reviewers. Any product that may be evaluated in this article, or claim that may be made by its manufacturer, is not guaranteed or endorsed by the publisher.
